# Revascularization and Apical Plug in an Immature Molar 

**DOI:** 10.22037/iej.v13i1.19666

**Published:** 2018

**Authors:** Leyla Roghanizadeh, Mahta Fazlyab

**Affiliations:** a * Dental Research Center, Research Institute of Dental Sciences, Dental School, Shahid Beheshti University of Medical Sciences, Tehran, Iran; *; b * Iranian Center For Endodontic Research, Research Institute of Dental Sciences, Dental School, Shahid Beheshti University of Medical Sciences, Tehran, Iran; *; c * Department of Endodontics, Dental School, Islamic Azad University, Tehran, Iran*

**Keywords:** Apexification, Apical Plug, Calcium-enriched Mixture Cement, CEM Cement, Endodontics, Regeneration, Revascularization

## Abstract

Managing of necrotic permanent teeth with immature apices is a treatment challenges. Treatment of such teeth includes apexification, apical plug and more recently, revascularization technique with the probable advantage of continuation of root development. In the present case report the referred patient had discomfort with a necrotic immature mandibular first molar. Periapical radiography showed a rather large apical lesion around immature roots. Revascularization protocol using calcium-enriched mixture (CEM) cement was indicated for the mesial root. However, in distal canal apical plug technique was applied. At 2-year follow-up, both procedures were successful in relieving patient’s symptoms. Dentin formation and increase in length of the mesial root was obvious. Apical plug and revascularization technique proved to be successful in management of necrotic immature teeth; moreover, revascularization carried the advantage of continuation of root development.

## Introduction

For years, apexification with calcium hydroxide (CH) as an apical barrier technique was the treatment of choice for immature necrotic teeth with incomplete roots and large apical foramina. However, long time for formation of apical barrier, multi-visit treatment sessions necessitating patient compliance and tooth fracture susceptibility are the disadvantages of this approach [1, 2]. 

The innovative concept of pulp regeneration in management of immature nonvital teeth have been noticed in recent decade [3]. Historically, vital tissue brought to existence in canal space following avulsion were milestones of using revascularization in immature teeth [4, 5]. The basis of the technique is migration of stem cells probably from apical papilla, having an appropriate blood coagulation as a scaffold and availability of growth factors that could be provided by platelets or dentin [3, 6, 7]. In 2011, the results of a molecular investigation proved that stem cell markers levels increased in blood samples collected from bleeding evoked in root canals of treated teeth with revascularization protocol compared to systemic blood; moreover, bleeding caused by periapical irritation would deliver stem cells into canal space which express markers of a subpopulation of mesenchymal stem cells [8]. Nowadays, discussion about histologic, radiographic and clinical outcomes and the degree of tissue formation in this modern technique, particularly in comparison to apical plug, is continuing.

With improvements in biomaterials, calcium-silicate cements such as mineral trioxide aggregate (MTA) and calcium-enriched mixture (CEM) cement are developed for regenerative procedures in endodontics. Both have been used as artificial root-end barriers and successful outcomes have been reported [1, 9, 10] .

**Figure 1:**
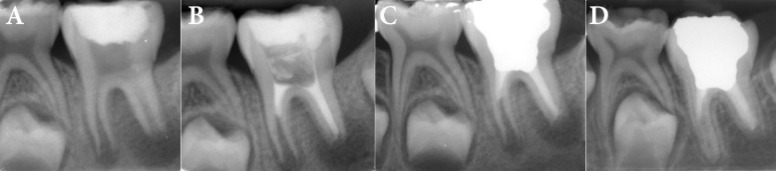
*A)* Permanent immature first molar, with chronic apical periodontitis, went under emergency treatment; *B)* Mesial canals treated by revascularization protocol; distal canal was treated using apical plug technique; *C)* Tooth was permanently restored with amalgam; *D)* two-year follow-up radiography: resolved periradicular lesions and continued development of mesial root

This report demonstrates management of an immature mandibular first molar with periradicular lesions where the mesial canals were treated by revascularization protocol and the distal canal underwent apical plug technique.

## Case Report

A 10-year old girl with chief complaint of pain in a left mandibular molar was admitted by a general dentist. The permanent mandibular first molar had extensive caries which was removed all the caries and emergency pulpotomy was done by the dentist. Then, the patient was referred to endodontic department of a private dental clinic. The medical history was non-contributory. In clinical evaluation, there was a significant swelling in the adjacent buccal vestibule. The tooth was sensitive to percussion/palpation. The probing depth of adjacent gingiva was normal, and there was no mobility. The tooth did not show any response to the different pulp tests. The radiographic examination (Figure 1A) revealed immature roots with open apices. A large radiolucent lesion around the distal root apex and smaller lesions at the apex of mesial root and at the furcal area were observed. The ultimate diagnosis was pulp necrosis with symptomatic apical periodontitis. Revascularization protocol was considered as the optimal treatment plan. It was explained to the parents and a written consent was obtained.

At the first treatment session, after local anesthesia using 3% plain mepivacaine (Scandonest, Septodont, Emu Plains, Australia) the tooth was isolated with rubber dam. The access cavity was completed by means of a high-speed handpiece and abundant water spray. The working length was measured by Root ZX apex locator (Morita, Kyoto, Japan). The root canals were gently irrigated and filled with 5.25% NaOCl and passively instrumented with K-files (Mani, Tochigi, Japan).

The canals were dried using paper points. Same proportions of ciprofloxacin, metronidazole and minocycline were mixed with saline to achieve a creamy paste. It was carried into the canals with a #25 K-file. The canals were filled with the paste 3 mm shorter than the working lengths up to CEJ. Then the access cavity was sealed with Cavit (ESPE-Premier, Norristown, PA, USA).

The second treatment session was arranged four weeks later, when signs and symptoms were healed, so there was no swelling and the tooth was not sensitive to percussion or palpation. To facilitate bleeding in revascularization procedure, local anesthesia with 3% plain mepivacaine (Septodont, Cedex, France) without any vasoconstrictor was done. Following isolation and removal of temporary restoration, the triple antibiotic paste was removed by irrigating and gently filing each canal, using 5.25% NaOCl. After drying the canals with paper points, a sterile #40 K-file in the distal canal and sterile #20 K-files in the mesial canals were passed through the apices to irritate periapical tissues. Bleeding was induced in all canals and was controlled below the CEJ level of each canal. After 10 min waiting for blood clot formation, CEM powder and liquid (BioniqueDent, Tehran, Iran) were mixed according to manufacturer’s instructions. The paste was carried to the canals and adapted with large sizes paper points. The pressure applied to place CEM in the distal canal was probably more than enough; therefore, the cement was pushed to the apical portion of the canal and built an apical plug there. Consequently, the distal canal filled with CEM thoroughly. In the mesial canals, the paste was fitted to the dentinal walls up to the CEJ, forming a 5-mm layer cement. Finally, the tooth temporized with Cavit (Figure 1B).

The day after, the temporary restoration was removed to check and confirm CEM setting. Then, permanent coronal restoration was done with amalgam (Cinalux; Faghihi, Tehran, Iran) (Figure 1C). In 1-month follow-up session, there was full recovery from all signs and symptoms. In 2-year follow-up radiographic evaluation (Figure 1D), complete healing of the apical lesions was evident. In the mesial root, additional dentin formation on the root walls and increasing in the root length was observed.

## Discussion

Managing of necrotic immature permanent teeth has been a debatable subject in endodontics [3]. In the present case there was endodontic success in treatments of all roots with both methods of apical plug and regenerative procedure. Moreover, revascularization resulted in continuation of root development. Calcium-silicate based cements such as MTA and CEM cement have been successfully employed in both procedures and there was no priority in successful rate between these protocols in healing periradicular lesions [11, 12].

Best quality scientific evidences are crucial to provide evidenced-based recommendations for clinical procedures [13]. High quality evidences such as randomized clinical trials indicating reliability of the regenerative endodontic procedure have not been generated yet [14]. Current studies complain less than enough evidence to prove efficacy and predictability of this method in continuing root development [11, 15, 16]. Moreover, it cannot be evidence-based indicated either apical plug or revascularization procedure would be more successful in curing necrotic immature teeth with apical periodontitis. Both methods are used in clinical practices; however, the edge of science moves towards regenerative protocols [14].

Randomized clinical trials have demonstrated reliable outcomes for apexification with CH and MTA [17], although comparative evaluations presented advantage of MTA in reducing treatment time [1, 18]. A recent case-series exhibited the success of CEM apical plug in managing teeth with open apices [10].

Many reports explained good prognosis subsequent to regenerative endodontic treatments including revascularization [3, 19-21]. The investigators recommended this procedure in conditions of immature teeth not being appropriate for canal obturation, poor results from traditional techniques such as apexogenesis, apexification or partial pulpotomy, and if the revascularization method can improve the tooth survival [22]. Some retrospective studies, comparing regenerative endodontic treatment and apical barrier technique, demonstrated no statistical difference in survival and clinical success between protocols [11, 15, 23]. There might be more incidences of adverse events and failure in revascularization group, as the teeth became re-infected needing another endodontic intervention [11]. Despite curing the infection, resolving the periapical lesions and successful clinical outcomes, revascularization may result in inconstant root development patterns [24]. Although some studies and reports have shown the anticipated root development following revascularization [3, 20, 21, 23]; other exhibited no significant increase in root radiographic measurements [11, 15, 16]. Probable thickening of the root walls may reinforce the roots against fracture, and may increase the survival of the treated tooth [23]. However, major biologic factors, characterization of developed tissue and long term results are uncertain and debatable [14, 25].

In this case, CEM cement has been used as both the coronal barrier in revascularization and the apical barrier in apical plug. Sealing ability of CEM cement is comparable to MTA [26, 27]. The material is biocompatible in terms of cytotoxicity and genotoxicity [28, 29] and demonstrated favorable outcomes in vital pulp treatments [30-34]. CEM cement has been applied in various endodontic, reparative, and regenerative procedures in dentistry such as apical plug [10, 20] and revascularization method [3, 20], achieving satisfactory results. The clinical and radiographic outcomes of the current case showed successful application of this biomaterial in both protocols. 

## Conclusion

Apical plug/revascularization with CEM might have success for managing necrotic immature permanent teeth.
